# Newman Neild

**Published:** 1934

**Authors:** 


### NEWMAN NEILD, M.B., Ch.B., F.R.C.P.

THE tragically sudden death of Dr. Newman Neild, in the early hours of 4th July, came as a great shock to his patients and friends, as he had been at work less than twelve hours before. There had been, however, certain misgivings in the minds of his professional colleagues during the previous few months, in spite of apparently undiminished energy.

Newman Neild was born in Bristol in 1872, and received his medical education at Owen's College, Manchester, where his father was Principal of Dalton Hall, one of the hostels of Owen's College. He took the degrees of M.B., Ch.B. (Vict.), with Honours, in 1896, and the M.R.C.P. (Lond.) in 1907, being elected a Fellow in 1928. After resident appointments at the Manchester Royal Infirmary, the Brompton Hospital for Consumption, and the Children's Hospital, Great Ormond Street, as well as Clinical Assistantships in Manchester and London, he finally settled in practice in Bristol. In 1901, when he was appointed Assistant Physician to the Bristol General Hospital, began a connection which was only broken by his untimely death. After eighteen years as Assistant Physician he was appointed to the full staff in 1918, and for the last seven years had been Senior Physician. His service to that institution was devoted indeed, and while he never shirked his ward-work, his Out-patient Department always seemed to come first in his affections. No out-patient who wished to see him was ever turned away, and no part of the heavy work entailed was ever delegated to a junior. For twenty-eight years he was Honorary Physician to the Clergy Daughters' School, and the care and time he devoted to the affairs of the school was immense. Nowhere will Neild be more missed than there. Neild was always an ardent supporter of medical education for women : in the early days, when women doctors were not taken as a matter of course, as is the case to-day, Neild was always ready and willing to have a woman House Physician. When a Consulting Physician was to be appointed to the Walker Dunbar Hospital—an institution entirely staffed by women, so far as the active staff was concerned—the choice naturally fell on Newman Neild, and he served there to the end. In his professional work Neild's faith in drugs was absolute. He was Lecturer in Pharmacology and Therapeutics in the University for many years, and Examiner there, as well as at Birmingham University and the Royal College of Physicians. He was an ardent supporter of the British Medical Association, being Secretary to the Bath and Bristol Branch from 1905 till 1923. He served the Bristol Division, both as Secretary and Treasurer, for many years, and was President of the Branch in 1923-24. He also acted as Representative in 1916-18. Apart from his professional work, Neild's interests were many. He was no mean draughtsman with the pencil, an ardent collector of china and pottery, a botanist of great experience, and an indefatigable collector of curios of all sorts. He had a very special knowledge of Bristol Delft, and his opinion was much sought after by experts from all over the country. Any spare holiday was spent scouring the downs of Wiltshire for flints, or the peat-moors of Somerset in search of botanical rarities. Neild's activity of brain was such that in conversation he would range from subject to subject with such speed that even the keenest-witted of his audience would be left bewildered. He was a practised raconteur, and his fund of stories was so extensive that he rarely retailed the same one twice.

What memory of this protean character will linger longest in the minds of those who knew him best at work and play ? Perhaps it will be his devotion to his out-patients at the General Hospital. There he inspired such devotion by his untiring efforts for his patients' welfare that they frequently tried to show their appreciation of his kindness and skill by making him presents, and at Christmas his table would often be covered by such expressions of regard. A last memory of Newman Neild must be of many hours spent in his company, and not one of them a dull one.

To his widow and two daughters will go out the very deep sympathy of friends and patients all over the West of England.

